# Curved Magnetic Hydrogels for Understanding Cancer Initiation

**DOI:** 10.1021/acsami.5c11408

**Published:** 2025-08-28

**Authors:** Ana C. Manjua, Christian M. Verkerk, Burcu Gumuscu

**Affiliations:** † Biosensors and Devices Lab, Department of Biomedical Engineering, 3169 Eindhoven University of Technology, Eindhoven 5600 MB, The Netherlands; ‡ Institute of Complex Molecular Systems, Eindhoven University of Technology, Eindhoven 5600 MB, Netherlands; § Eindhoven Artificial Intelligence Systems Institute, Eindhoven University of Technology, Eindhoven 5600 MB, The Netherlands

**Keywords:** curved scaffold, magnetic scaffold, polyacrylamide, magnetic nanoparticles, MDA-MB-231 breast cancer cells, NHDF dermal fibroblasts, cancer cell dynamics

## Abstract

Cancer development is increasingly associated with changes in tissue curvature, which influence dynamic cellular behaviors such as polarization and migration. However, the mechanisms by which curved tissue architectures contribute to cancer progression remain poorly understood, partly due to the lack of adequate research tools. Here, we fabricated magnetic Acrylamide hydrogel constructs to investigate the effect of curvature and dynamic movement induced by external magnetic fields of low intensity (100 mT) in the phenotype and gene expression of MDA-MB-231 metastatic breast cancer cells. We found that combining the magnetic hydrogel (MACrylamide) with an applied magnetic field significantly reduced the area occupied by the cells, from 55% to 33%, compared to conditions without magnetic field exposure. In addition, exposure to the magnetic field in combination with the magnetic scaffold had a statistically significant effect on the expression of specific genes associated with anti-inflammatory responses and tumor proliferation and metastasis (NMO1, OCT4, SOX4). These findings suggest that the cells altered their behavior after 7 days of culture on the magnetic hydrogel and 24 h of magnetic stimulation. As a control, NHDF dermal fibroblasts were cultured under the same conditions. Comparison of the two cell lines confirms the selectivity of the approach for cancer cells while ensuring minimal impact on healthy skin cells. This work underscores the importance of dynamic tissue curving during carcinogenesis using a biocompatible surface that mimics physiologically relevant curved tissues.

## Graphical abstract

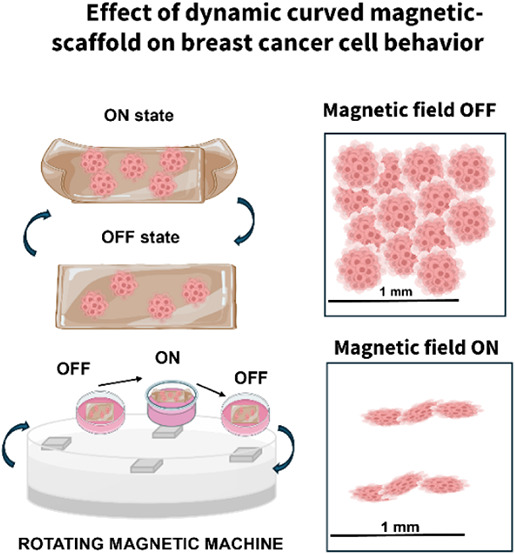

## 1 Introduction

Like any other neoplasm, cancer development is a complex multistage process with a high level of molecular and morphological heterogeneities.^1^ During this development, cells continually experience biomechanical deformations which ultimately lead to the formation of curved portions of tissue. Biomechanical deformations are dynamic in nature and influence the behavior of cells, which in turn contribute to tissue formation.^2^ Still, the curvature of tissue surfaces is considered to contribute to cancer initiation, as mutated cell growth disrupts normal epithelial structure during cancer development.^3,4^ Nonetheless, the biophysical parameters underlying the formation of abnormal tissue shapes remain unknown, mostly due to the focus of understanding cancer progression through conventional 2D and 3D cell culture systems and animal models.^5^ While complex animal models made it difficult to identify the source of the stimuli, simple cell culture approach failed to mimic physiologically relevant dynamic mechanical conditions.^5^ The possibility to construct physiologically relevant materials able to alter their shape dynamically can have a big impact on the design of novel cellular therapies and the advancement of *in vitro* testing methods. To understand the impact of curvature on cancer models, the main bottleneck is the mimicking of microscale-range dynamic curvatures (curve radius in the μm range) that can be sensed by cells.^6,7^

In recent years, various types of mechano-responsive hydrogels have been engineered for biomedical applications. These include hydrogels designed for friction-based damping, which effectively dissipate mechanical stress to promote rapid material recovery after deformation and protect encapsulated cells from mechanical trauma during repetitive compression.^8^ Temperature-sensitive hydrogels based on the nonfunctionalized benzene-1,3,5-tricarboxamide supramolecular system have also been developed, enabling 3D cell encapsulation through tunable mechanical properties.^9^ Additionally, strain-stiffening hydrogels have been employed to replicate the mechanical behavior of biological tissues, serving as artificial tissues, scaffolds, or wound dressings. Their shear-thinning properties allow for injectability and minimally invasive, region-specific delivery.^10,11^ Mechanochromic hydrogels have further enabled real-time visualization of mechanical stress, with promising applications in biosensing and diagnostics.^12^

Recent studies have highlighted the role of tissue curvature as a biomechanical cue that can influence cancer initiation and progression,^13^ in particular breast cancer.^14^ Curvature-induced changes in cell shape and membrane tension have been shown to modulate intracellular signaling pathways and transcriptional programs associated with malignancy. For example, curvature can regulate the epithelial-mesenchymal transition (EMT), a key process in cancer metastasis, by altering cell polarity and cytoskeletal organization.^15^ Additionally, curvature affects actin flow and cell motility, with higher curvature leading to reduced spreading and migration efficiency in confined environments.^16^ Regarding breast cancer cells response to mechanical deformations, some authors have concluded that breast cancer cells (both MCF-7 and MDA-MB-231 cells) can modify their mechanical properties in response to the stiffness of the surrounding microenvironment or 3D scaffolds.^17–20^ These findings suggest that curvature is an active regulator of cellular behavior, capable of influencing cancer cell fate through mechanotransduction and signaling pathway modulation.

Furthermore, authors have established a connection between magnetic actuation and breast cancer cell growth. The study investigated the antiproliferative effect of preincubating breast cancer cells (MCF-7) with iron oxide nanoparticles combined with static magnetic field exposure. This led to increased doxorubicin-induced cytotoxicity and apoptotic cell death induction.^21^ Another work exposed these cells to static or 50 Hz MFs at 100 μT with or without chemotherapeutic drug for 3 h, showing that magnetic actuation induced modifications to doxorubicin treatment, but the survival of the doxorubicin-treated MCF-7 cells was otherwise unaffected.^22^ Using MDA-MB-231 breast cancer cells, authors reported viability changes and decreased adhesion with altered pathways using low-frequency magnetic fields, but increased invasion and migration capacity.^23^

Following these controversially interesting works, the present study focuses on the carcinogenic impact of dynamic, magnetically responsive curved surfaces, which mimic tissue deformations associated with tumor initiation and progression.

We selected breast cancer as a model system due to its well-characterized progression from ductal carcinoma in situ (DCIS) to invasive ductal carcinoma (IDC), a transition often accompanied by changes in tissue architecture and mechanical microenvironment.^24^ Specifically, we used the MDA-MB-231 cell line, a widely used model of triple-negative breast cancer (TNBC), which represents one of the most aggressive and mechanosensitive subtypes of breast cancer.^25^ MDA-MB-231 cells are characterized by their mesenchymal phenotype, high motility, and pronounced responsiveness to biomechanical cues such as substrate stiffness, confinement, and curvature.^26^ These properties make them particularly suitable for investigating how topographical features—like curvature—can influence cellular behavior and potentially initiate malignant transformation. By leveraging this model, we aim to explore the mechanobiological mechanisms that may underlie early cancer progression in geometrically complex tissue environments.

We developed MACrylamide, a hydrogel scaffold embedding 3% (v/v) iron oxide nanoparticles in polyacrylamide, capable of reversible, micrometer-scale indentation and expansion through magnetic actuation. Characterization showed MACrylamide’s stiffness (∼98 kPa) matches malignant breast tissue, which has been chosen as model disease in our work. MACrylamide also showed an increased surface roughness and magnetically induced wettability changes compared to regular Acrylamide. Aggressive metastatic breast cancer cells (MDA-MB-231) showed a 22% reduction in cell area under the combined effect of magnetic hydrogel and magnetic stimulation and a ∼600-fold downregulation of oxidative stress marker NMO1 without stimulation, while stemness genes were upregulated by static curvature. Healthy fibroblasts remained largely unaffected. These findings demonstrate that MACrylamide’s combined curvature and magnetic actuation selectively modulate cancer cell behavior through mechanotransduction and oxidative stress responses, while sparing healthy fibroblasts. This highlights the increased mechanosensitivity of cancer cells and suggests our platform’s potential for dissecting mechanobiological regulation in tumor progression. Ultimately, MACrylamide enables the study of cell–material interactions under (i) external magnetic fields serving as a simple, repeatable, and noninvasive stimulus; and (ii) dynamically curved hydrogels that generate physiologically relevant mechanical cues. While the influence of magnetic fields on cancer cell behavior remains largely underexplored,^27–31^ MACrylamide offers a reproducible framework to study these effects *in vitro*.

## 2 Results and Discussion

### 2.1 Characterization of the Magnetic Responsiveness of the Material

Acrylamide-based hydrogels have long been used as 2D and 3D cell culture scaffolds. These hydrogels can have their mechanical properties tuned based on the dosages and binding densities of biomacromolecules.^32–35^ Their stiffness can also be adjusted by changing the amount of monomer and cross-linker during their UV-induced free radical polymerization.^36^ In this study, we synthesized MACrylamide, a composite hydrogel consisting of polyacrylamide embedded with 3% v/v iron oxide magnetic nanoparticles. Prior to casting the hydrogel, the glass substrate was patterned with a silane solution to promote adhesion and facilitate curvature upon cross-linking (Figure 1A).

**Figure 1:**
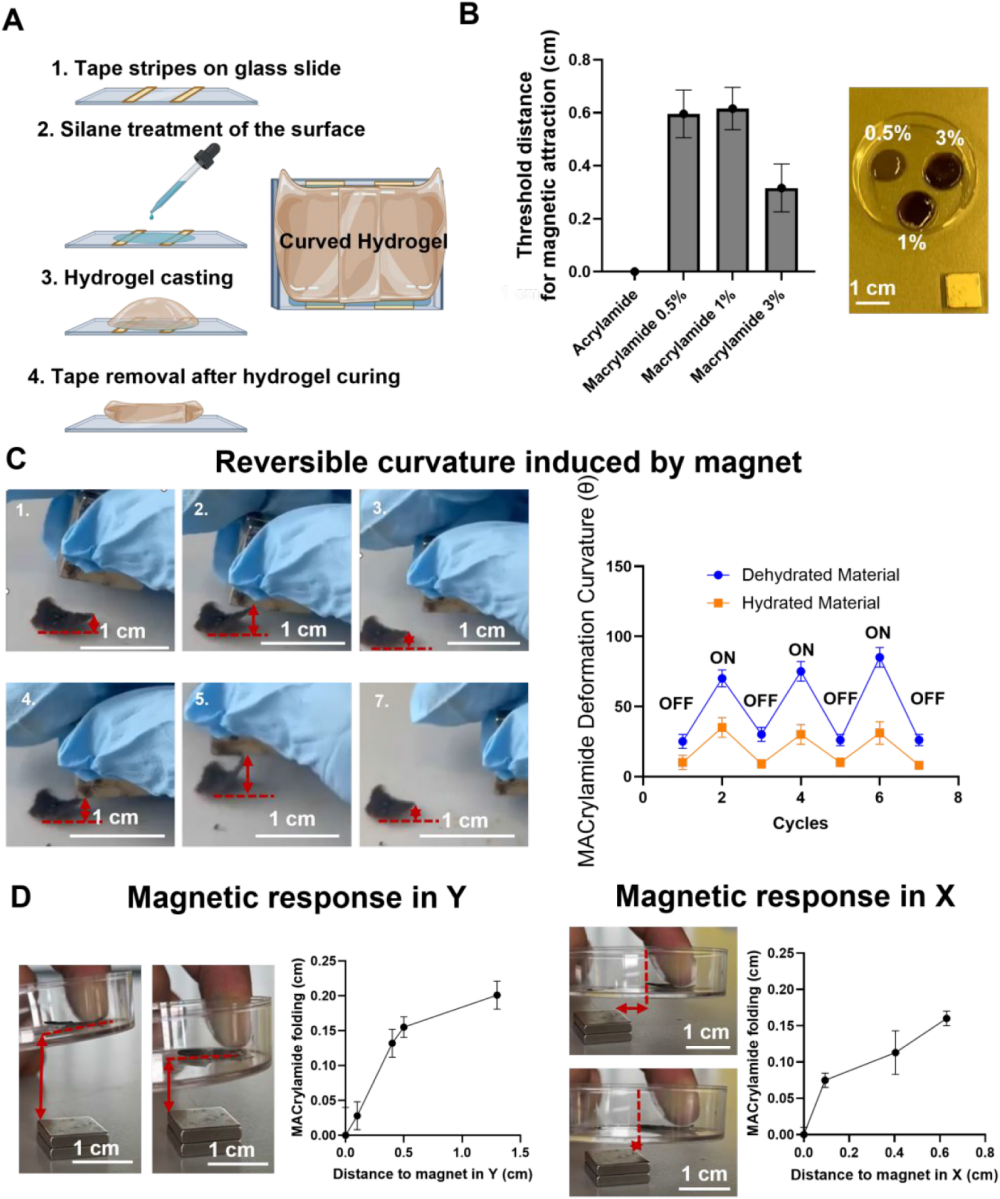
Magnetic responsiveness of MACrylamide. (A) Schematic representation of the fabrication process for the curved magnetic hydrogel. (B) Minimum distance required for magnetic attraction as a function of MNP concentration in acrylamide hydrogels (0.5%, 1%, and 3% v/v), *n* = 3. (C) Reversibility of curvature in dehydrated and hydrated 3% MACrylamide during repeated magnetic ON/OFF cycles (*n* = 30). (D) Characterization of maximum folding as a function of distance to the magnet along the *X* and *Y* axes (*n* = 3). Data presented as means ± SD.

To assess the magnetic responsiveness of the material, we prepared Acrylamide hydrogels with varying magnetic nanoparticle (MNP) concentrations (0.5%, 1%, and 3% v/v) and measured the threshold distance at which a neodymium magnet (100 mT maximum field strength) could induce bending (Figure 1B). The hydrogel containing 3% MNP exhibited a bending response at 0.35 cm, while hydrogels with 0.5% and 1% required a closer proximity of 0.6 cm to show detectable magnetic attraction. As a control, a nonmagnetic Acrylamide hydrogel was also tested and as expected, showed no magnetic response. A similar test was previously performed using a magnetic κ-carrageenan–collagen bioink,^37^ where the minimum distance required for magnetic attraction ranged from 2 to 6 cm. In contrast, MACrylamide responds at much shorter distances, likely due to its lower cross-linking density, which permits greater mobility of magnetic particles within the hydrogel matrix.

To characterize the potential for reversible curvature, we performed three cycles of magnet sliding over both hydrated and dehydrated formats of MACrylamide (3% v/v MNP) to determine the maximum achievable curvature after repeated actuation (Figure 1C). During the magnetic field ON cycles, dehydrated format consistently achieved bending angles between 85° and 95°, while hydrated format reached approximately 43°. In the OFF cycles (i.e., without magnetic stimulation), the curvature partially relaxed, returning to about 24° in dehydrated format and 12° in hydrated format. Overall, the dehydrated format exhibited a greater degree of responsiveness compared to the hydrated ones, likely due to the increased weight of the swollen hydrogel, which may hinder its ability to bend toward the magnetic field.

To further characterize magnetic responsiveness, we tested actuation along both the *X* and *Y* axes to simulate the multidirectional forces applied during cell culture experiments (Figure 1D). Along the *Y*-axis, a partial folding of 0.19 cm was observed at a magnet distance of 1.6 cm, which was considered the baseline curvature. Complete folding of the dehydrated hydrogel occurred when the magnet was directly aligned along the *Y*-axis. A similar response was seen along the *X*-axis, where the hydrogel gradually folded as the magnet approached. Together, these findings demonstrate that MACrylamide exhibits strong and reversible magnetic responsiveness in both hydrated and dehydrated states.

Finally, to assess batch-to-batch reproducibility, curvature radius was quantified from *n* = 5 samples per batch using ImageJ. Three independent batches were analyzed, each prepared under identical conditions. The mean curvature radius for MACrylamide was found to be 2.2 ± 0.17 cm, with no statistically significant difference between batches (*n* = 5, ANOVA, *p* > 0.05), indicating consistent fabrication. Acrylamide hydrogels were also analyzed for radius curvature (4 ± 0.17 cm), evidencing an expectedly larger curvature radius corresponding to a flatter surface, as shown in Figure S1.

### 2.2 Characterization of the Curved Hydrogel Properties

A thorough characterization of the physical, mechanical, and surface properties of the MACrylamide hydrogels was sought to assess their suitability as dynamic biomaterials. These baseline evaluations included morphological and mechanical profiling, swelling behavior, cytocompatibility, and magnetically induced wettability changes. Together, these experiments establish a comprehensive understanding of the material’s performance under conditions relevant to the upcoming biological assays.

Based on the results from Figure 1, the 3% (v/v) MNP concentration demonstrated superior magnetic responsiveness in comparison with MACrylamide with 1% and 0.5% of magnetic nanoparticle loading. Acrylamide hydrogels without MNPs were used as controls throughout the study. Figure 2A shows a schematic and macroscopic view of MACrylamide and control Acrylamide (0% MNPs), alongside scanning electron microscopy (SEM) images of their surfaces. The SEM analysis revealed that MACrylamide exhibits noticeably higher surface roughness (average particle size on the surface = 3.5 ± 1.3 nm) compared to the smoother surface observed in the control sample. This is an expected result due to the presence of MNPs in MACrylamide.

**Figure 2:**
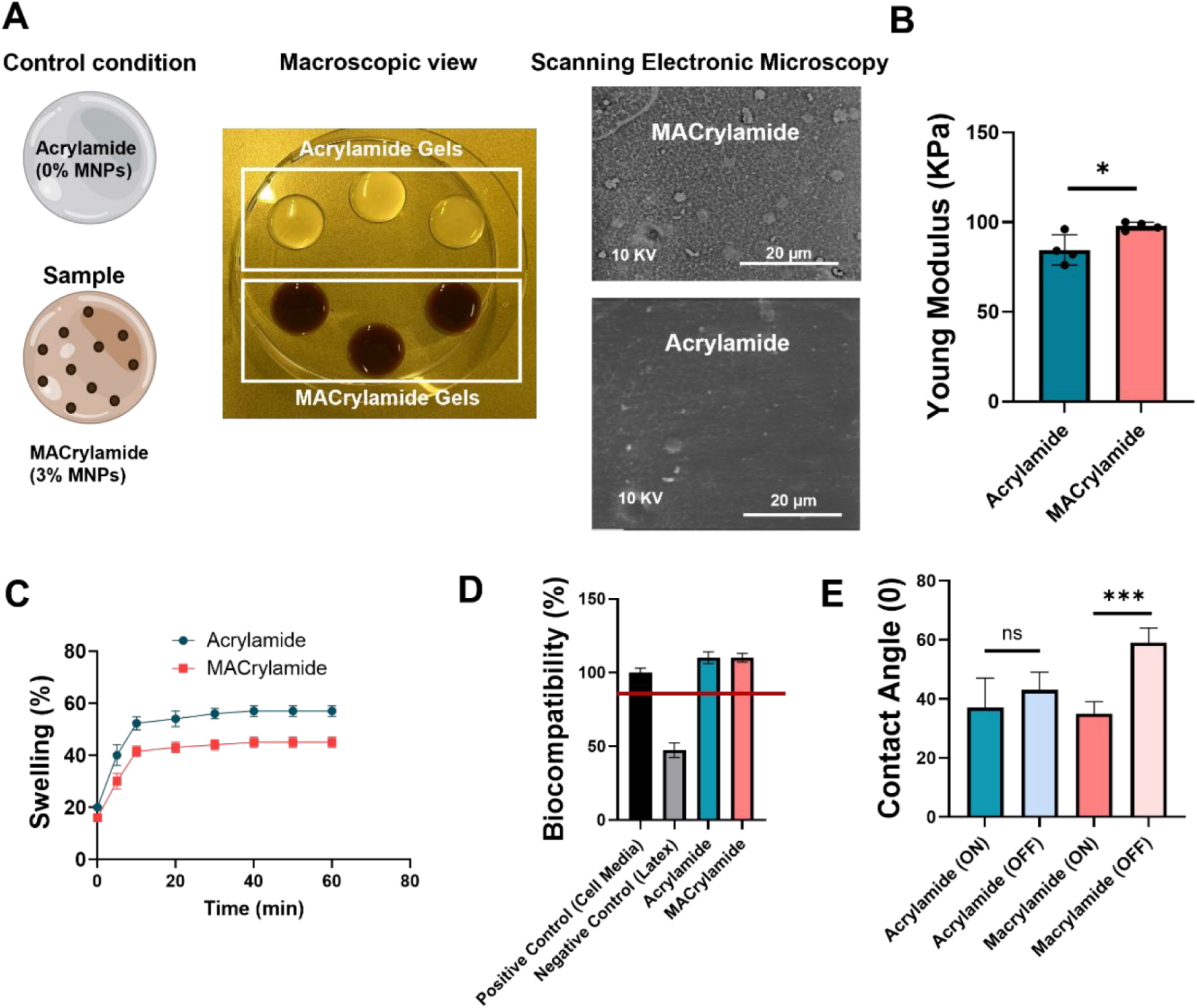
Characterization of the properties of the MACrylamide and Acrylamide. (A) Representation of MACrylamide (3% v/v MNPs) and Acrylamide (0% v/v MNPs) in a schematic view, macroscopic and surface observation using SEM (B) Mechanical characterization of MACrylamide and control using compression testing (*n* = 5). (C) Swelling rate of MACrylamide and Acrylamide (*n* = 3). (D) Assessment of the biocompatibility of the hydrogels in comparison with a positive control (cells cultured in cell media) and negative control (cells cultured with the lixiviates of latex material) using MTT assay (*n* = 3). (E) Surface wettability characterization of the hydrogels via contact angle measurements (*n* = 5). Data presented as means ± SD.

Mechanical characterization was performed via compression testing to determine the Young’s modulus of each hydrogel. The control Acrylamide exhibited a Young’s modulus of 84.5 ± 7.3 kPa, while MACrylamide displayed a slightly higher modulus of 97.8 ± 1.9 kPa (Figure 2B). Representative stress–strain curves are shown in Figure S2. According to the literature, the elasticity of healthy breast tissue (from adipose to fibroglandular tissues) typically ranges from 3.25 to 16 kPa, whereas breast tumor tissues, particularly those associated with invasive ductal carcinoma or ductal carcinoma *in situ*, have reported Young’s moduli between 18 and 94 kPa.^38^ A review study^39^ reports several works confirming that the increased stiffness of these pathological tissues is often associated with tumor type and aggressiveness. Given that this study uses metastatic MDA-MB-231 cancer cells (one of the most aggressive subtypes) the stiffness of MACrylamide falls within the upper range reported for malignant breast tissues, confirming its mechanical suitability for the intended biological studies.

The swelling properties of the hydrogels were also evaluated (Figure 2C), showing that the incorporation of MNPs slightly reduces the water absorption capacity of the hydrogel. MACrylamide reached a maximum swelling capacity of 45%, compared to 58% for the Acrylamide control. This decrease is likely due to the volume occupied by the magnetic particles, which decreases the available free space within the polymer network for water uptake. A similar effect has been reported in magnetic hydrogels composed of gelatin.^40^

To evaluate the biocompatibility of the hydrogels for future cell studies, an MTT assay was conducted using dermal fibroblasts (Figure 2D), with latex and culture medium serving as negative and positive controls, respectively. Both Acrylamide and MACrylamide supported high levels of cellular metabolic activity, exceeding 80% and reaching approximately 110% relative to the positive control. These findings align with previous work from our group demonstrating the compatibility of similar magnetic Acrylamide-based hydrogels.^27^ It is important to note that both materials showed cytotoxic effects prior to a washing step designed to remove unbound, potentially toxic compounds. The biocompatibility of MACrylamide samples with varying MNP concentrations was also assessed through MTT assay at 0.5%, 1%, and 3% MNP loading. The results (Table S1) showed no significant cytotoxicity differences among the tested concentrations, indicating that all formulations are suitable for biological applications. Given the enhanced magnetic responsiveness observed at 3% MNP, this concentration was selected for subsequent experiments. In addition, a complementary assay to the MTT test confirmed the absence of inhibition halos after 24 h of direct contact with the hydrogels, further supporting their cytocompatibility (Figure S3).

Contact angle measurements under magnetic field ON and OFF conditions revealed a statistically significant decrease for MACrylamide, from 59° (OFF) to 35° (ON), indicating increased surface hydrophilicity upon magnetic stimulation (Figure 2E). Interestingly, the contact angle of MACrylamide under magnetic field ON conditions was similar to that of the Acrylamide control, which remained unchanged between ON and OFF modes. This behavior is characteristic of magnetic hydrogels and is attributed to the changes in surface roughness upon magnetic exposure, as previously reported for poly­(vinyl alcohol) and gelatin-based hydrogels.^28,40^ Given that our cell experiments are subjected to a rotating magnetic device, it is important to emphasize that cells on MACrylamide experience not only dynamic changes in curvature at a frequency of 0.2 Hz (as characterized in Figure 1), but also cyclical alterations in surface wettability during the exposure to the external magnetic field (Figure S4). In addition, and according to the Maxwell–Faraday Law, the oscillatory nature of this field is responsible generation of an electrical field, already described in the literature to interfere with biomolecule and ion interactions in cell surface recognition mechanisms.^29^ Nonetheless, the temperature of the cell culture media was measured overtime during magnetic exposure and found to be stable at 37 °C.

### 2.3 Effect of the Curved Hydrogel on Cancer Cell Behavior

The main objective of this study is to investigate how the curvature and surface dynamics of MACrylamide hydrogels influence the behavior and fate of cancer cells. We selected metastatic MDA-MB-231 breast cancer cells as a model due to their aggressive phenotype and well-characterized role in studies of neoplastic transformation and metastatic progression.^4,6^ *In vitro* studies using breast cancer cell lines have long served as essential tools for exploring early diagnostic markers, testing therapeutic strategies, and studying cancer progression.^5^ Despite these advances, the effects of magnetic fields on cancer cells remain controversial, with studies reporting inconsistent impacts on proliferation, apoptosis, gene expression, and secretory pathways.^26–30^ This variability underscores the need for controlled model systems—such as magnetically responsive, curving hydrogels—to isolate and analyze the role of biomechanical and magnetic cues in cancer cell behavior. Magnetic fields are well established in clinical diagnostics (e.g., MRI), and magnetic nanoparticles have been investigated for targeted therapies such as hyperthermia, drug delivery, and nanoscale manipulation. Additionally, magnetic exposure is thought to influence oncogene expression by increasing reactive oxygen species and oxidative stress, although a direct link to cancer initiation has not been conclusively demonstrated.^41^

We began by assessing the biocompatibility of MACrylamide with MDA-MB-231 cells by culturing them directly on the hydrogel and comparing their behavior to cells grown on standard polystyrene surfaces (Figure S5). In addition, a Live/Dead assay was also performed with the MDA-MB-231 cells cultured on top of each scaffold to indicate good viability of the cells before magnetic actuation (Figure 3A).

**Figure 3:**
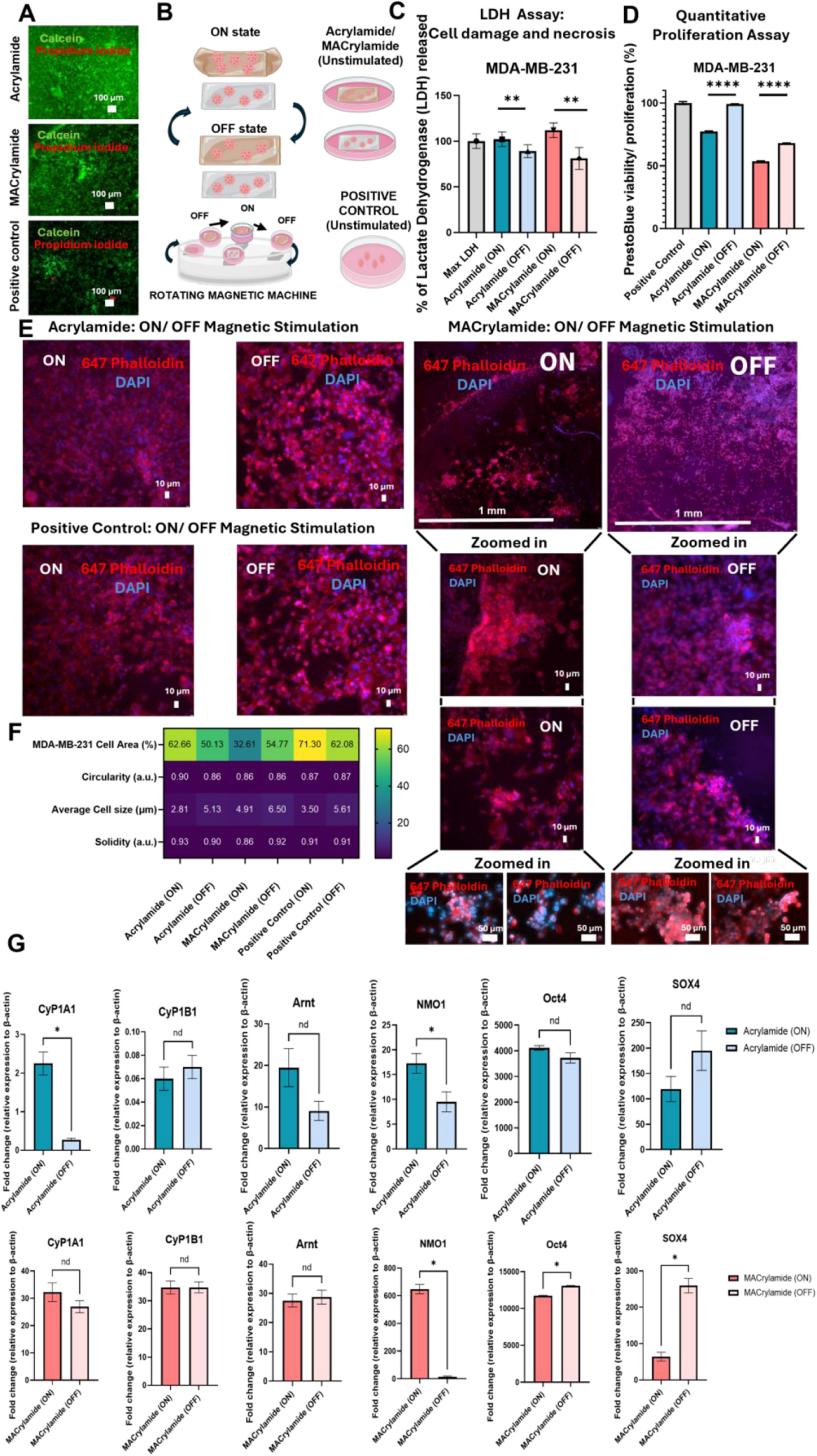
Effect of MACrylamide on breast cancer cells (MDA-MB-231). (A) Live (green)/dead (red) assay using MDA-MB-231 cells to show cell viability after seeding on top of Acrylamide and MACrylamide scaffolds and on polystyrene (positive control ). (B) Schematic representation of the magnetic stimulation setup and dynamic curvature changes under stimulation. (C) Quantitative LDH assay results of MDA-MB-231 cells on the final day of the experiment (day 7) for all tested conditions (*n* = 3). (D) Quantitative PrestoBlue viability and proliferation results of MDA-MB-231 cells on the final day of the experiment (day 7) for all tested conditions (*n* = 3). (E) Fluorescence staining of nuclei (DAPI, blue) and actin cytoskeleton (Phalloidin, red) in MDA-MB-231 cells cultured on MACrylamide (ON and OFF), Acrylamide (ON and OFF), and polystyrene (control) on day 7. (F) Heat map of MDA-MB-231 morphological features for all tested conditions (*n* = 3). (G) Relative gene expression of cancer-related genes for MACrylamide (ON and OFF) and Acrylamide (ON and OFF) on day 7 (*n* = 3). Data presented as means ± SD.

Microscopic imaging showed confluent cell layers with similar morphology across both conditions, as well as the positive control without scaffold, indicating that MACrylamide does not present any apparent compatibility issues. For this experiment, the MACrylamide hydrogel was fabricated to remain flat in the absence of a magnetic field (OFF) and to curve upon magnetic activation (ON), simulating dynamic physiological processes, while the Acrylamide scaffold remains flat under ON and OFF cycles of magnetic actuation. Figure 3B provides a schematic of the experimental setup, illustrating the magnetically induced curvature transitions controlled by a rotating magnetic device, as well as the condition using the flat scaffold of Acrylamide. We also involved polystyrene-cultured control groups with and without scaffolds of Acrylamide and MACrylamide without magnetic actuation (OFF) in all experiments for comparison. MDA-MB-231 cells were cultured on MACrylamide and Acrylamide for 7 days, with magnetic actuation applied for 24 h on day 6 using a rotating magnetic device. To assess potential effects on cell viability and stress, we performed a quantitative LDH assay on day 7 (Figure 3C). LDH (lactate dehydrogenase) release is commonly used as an indicator of cell membrane damage, necrosis, or apoptosis.^42^ However, in cancer cells, elevated LDH levels can also reflect enhanced glycolytic activity due to upregulation of LDH-A, which converts pyruvate to lactate. This can lead to increased intracellular LDH that may be released into the extracellular space due to metabolic shifts or altered membrane permeability, even when cells remain viable.^43^

Compared to the control condition (MDA-MB-231 cells cultured on polystyrene), we observed statistically significant differences in LDH release across conditions. The MACrylamide OFF group showed the lowest LDH release (81%), while the MACrylamide ON condition showed the highest (112%). Acrylamide ON and OFF groups exhibited intermediate levels at 102% and 89%, respectively. These findings suggest that the combined effect of magnetic field exposure and MACrylamide’s dynamic surface properties imposes increased stress on cancer cells, potentially contributing to cell death or metabolic reprogramming. Notably, the magnetic field alone also appears to influence cell behavior, as seen in the elevated LDH levels in the Acrylamide ON group and also observed in our previous findings.^27–29^ In Figure 3D, MDA-MB-231 cells were evaluated at experimental day 7 for their viability/proliferation properties using PrestoBlue assay. The results showed reduced viability/proliferative behavior for cancer cells simultaneously seeded on MACrylamide and exposed to magnetic actuation in comparison with the positive control and with cells seeded on Acrylamide.

To further investigate this behavior, next we performed morphological analysis to determine whether the observed biochemical changes were accompanied by structural alterations at the cellular level. We examined morphological changes using phalloidin-546 (red) for actin filament staining and DAPI (blue) for nuclear staining (Figure 3E). Cells cultured on Acrylamide (ON and OFF) and polystyrene (ON and OFF) showed only slight variations in size, morphology, and fluorescence intensity. In contrast, cells exposed to the magnetic field—regardless of substrate—appeared smaller, more elongated, and exhibited reduced fluorescence intensity, suggesting that magnetic stimulation alone can subtly influence cytoskeletal organization and cell morphology, which is an effect already demonstrated in the past for noncancerous cells.^28,29,44–46^

The most remarkable effects on cell morphology and distribution were observed when MACrylamide was used. To assess how dynamic curvature and surface properties influence cancer cell organization, we compared the MACrylamide ON and OFF groups. Under magnetic stimulation (MACrylamide ON), cells showed a marked reduction in surface coverage, with increased clustering and a greater proportion of empty regions on the hydrogel. In contrast, the MACrylamide OFF condition supported a highly confluent and evenly distributed cell layer. Zoomed-in images confirmed the more compact clustering and reduced fluorescence intensity in the MACrylamide ON group. Upon closer inspection, these cells appeared smaller, with a predominance of blue fluorescence, suggesting nuclei occupy a larger proportion of the cell body. In contrast, the MACrylamide OFF condition exhibited stronger red fluorescence, indicating a more extensive actin cytoskeleton and larger overall cell size in comparison with the ON conditions. This difference may be attributed to the absence of magnetic forces in the OFF condition, which otherwise distort the cells by unnaturally stretching them and reducing their native shape and area, as discussed before.^44–46^ Additional representative images for both conditions are provided in Figure S6.

To quantify these observations, we performed image-based morphometric analysis, summarized as a heat map in Figure 3F. These included measurements of total occupied cell area, circularity (how closely a cell approximates a perfect circle), average cell size, and solidity (a metric of perimeter smoothness or irregularity). Circularity values closer to 1.0 are generally associated with round, less motile cells,^47^ while lower solidity values (typically between 0.7–0.9) reflect irregular edges characteristic of invasive phenotypes.^44^ To determine whether magnetic field exposure alone could influence cell behavior, we also analyzed Acrylamide and polystyrene control conditions with and without magnetic stimulation. For Acrylamide, the total cell area decreased slightly from 62.7% to 50.1%, circularity dropped from 0.90 to 0.86, cell size increased from 2.8 μm to 5.1 μm, and solidity declined from 0.93 to 0.90. For polystyrene, the total area decreased from 71.3% to 62%, with cell circularity remaining stable at 0.87, size increasing from 3.5 μm to 5.6 μm, and solidity holding around 0.91.

The MACrylamide conditions showed more pronounced and distinct changes compared to Acrylamide results. When comparing OFF to ON, the total occupied area decreased from 54.8% to 32.6%, cell size shrank from 6.5 μm to 4.9 μm, and solidity dropped from 0.92 to 0.86, while circularity remained steady at 0.86. Taken together, these findings indicate that while magnetic stimulation alone has a mild impact on cancer cell morphology, the combination of MACrylamide and magnetic actuation produces stronger, statistically significant effects. These include reductions in cell-covered area and size, and changes in solidity. These results suggest that the cells may adopt a more compact and potentially aggressive profile in response to dynamic mechanical and surface cues. Taken together, these findings suggest a small but detectable influence of the magnetic field alone on cancer cell morphology. However, the most pronounced effects were observed when combining MACrylamide with magnetic stimulation. This condition led to a significant reduction in cell-covered area and size, and changes in solidity that could reflect a shift toward a more aggressive cancer cell profile in the OFF condition.

To further elucidate how the dynamic curvature and magnetic properties of MACrylamide affect breast cancer cell behavior, we conducted gene expression analysis focusing on markers related to oncogenesis, metastasis, stemness, and oxidative stress. An initial set of genes associated with inflammation, metabolism, stemness, and tumor progression was selected. GAPDH was first tested as a housekeeping gene for normalization but showed instability across conditions. We attribute this to its sensitivity to metabolic stress, oxidative responses, and apoptosis^48^ and therefore it was found unsuitable for studies involving magnetic exposure. Beta-actin, although known to be influenced by substrate stiffness and topography,^49^ showed more stable expression across conditions and was used for normalization. To account for its variability, gene expression comparisons were made within each material type (Acrylamide and MACrylamide), focusing on magnetic field ON vs OFF conditions (Figure 3G). The selected panel of genes included CyP1A1 (its upregulation is linked to pro-oncogenic effects such as enhanced cell migration and invasion;^50^ CyP1B1 (commonly overexpressed in breast tumors and associated with poor prognosis, increased invasiveness, and migration;^51^ Arnt (involved in regulating metabolism, survival under hypoxia, and potentially epithelial-to-mesenchymal transition;^52^ NMO1 (associated with increased proliferation and metastasis and commonly overexpressed in aggressive cancers;^53^ Oct4 (a marker of cancer stemness, self-renewal, tumor initiation, therapy resistance, and aggressiveness;^54^ and SOX4 (linked to cell motility, invasion, stemness, and activation of epithelial-to-mesenchymal transition.^55^

Our findings show overall higher upregulation of all tested genes in MACrylamide (both ON and OFF conditions) compared to Acrylamide (magnetic field ON and OFF), suggesting increased cellular activation on the magnetic scaffold. Only SOX4 and Oct4 were significantly upregulated in the MACrylamide OFF group, suggesting a potential shift toward a less differentiated or more plastic state in the absence of mechanical stress. While this observation raises the possibility of early stem-like transcriptional changes driven by scaffold conditions, further functional validation is required to confirm stemness acquisition. In previous studies, metastatic cells have been described as softer than nonmalignant cells, potentially due to increased compressive stress, tumor volume expansion, and stiffening of the extracellular matrix. This enhanced deformability of both the cell and its nucleus, along with the ability to adapt to successive microenvironmental constraints, is considered advantageous for metastatic potential.^56–58^ Therefore, our observed differences between MACrylamide ON and MACrylamide OFF conditions may reflect the adaptive response of MDA-MB-231 cells to dynamic magnetic actuation, coupled with the mechanical stimulation resulting from MACrylamide-induced shifts in curvature. For this reason, magnetic stimulation appeared to induce a stress response rather than increased malignancy. CyP1A1 was downregulated in both Acrylamide (2-fold) and MACrylamide OFF conditions, possibly reflecting reduced metabolic or detoxification demand without stimulation. Similarly, NMO1 expression dropped dramatically (by ∼600-fold) in MACrylamide OFF, indicating that oxidative stress signaling may be suppressed in the absence of magnetic actuation. These observations imply that magnetic actuation triggers oxidative stress responses, a phenomenon reported in prior studies involving magnetic stimulation of cancer cells.^59,60^ However, Arnt and CyP1B1 expression remained unchanged across all conditions, which may indicate that these genes are less sensitive to short-term magnetic or mechanical stimuli or that their regulation requires additional environmental factors or longer exposure times.

Taken together, these findings suggest a complex interplay between scaffold-induced mechanical cues and magnetic stimulation in regulating cancer cell gene expression and phenotype. While MACrylamide’s curved topography alone promotes gene expression patterns associated with stemness and potentially increased malignancy, the application of magnetic fields induces oxidative stress and metabolic responses that do not necessarily translate into increased aggressiveness. This is further supported by the observed reduction in cell area and lack of Oct4 and SOX4 upregulation under magnetic actuation, pointing toward a phenotype that may be more metabolically reactive but less proliferative.

To further investigate the role of the magnetic field and scaffold architecture on gene expression, we included a Positive Control group consisting of cells cultured on standard polystyrene tissue culture plates under ON and OFF magnetic actuation conditions (Figure S7). Notably, the downregulation of SOX4 and OCT4 and upregulation of NMO1 in the Positive Control ON condition, indicates that the magnetic field alone may influence gene expression even in the absence of active actuation. Even so, the gene expression profile in this control group closely resembled that of the acrylamide samples, suggesting a more dominant influence of the MACrylamide scaffold in the observed transcriptional changes, highlighting the synergistic effect of magnetic actuation and scaffold curvature.

A previous study using both human MDA-MB-231 and MCF-7 cells showed over 30% inhibition of breast cancer growth in mice by exposing the cells to a rotating magnetic field with intensities ranging from 0 to 0.15T and a low frequency of 4.2 Hz. It was referred by the authors that the same effect was not observed for other cancer types such as human gastrointestinal stromal tumor GIST-T1,^61^ although other studies have obtained promising therapeutic responses with magnetic stimulation for neuroblastoma or even neurodegenerative diseases.^62,63^ The observed downregulation of stemness associated with genes OCT4 and SOX4 in response to magnetic field stimulation may be mediated by mechanotransduction pathways that interface with transcriptional regulation. Previous studies have shown that magnetic fields, in particular oscillatory magnetic fields, can activate intracellular signaling cascades such as the MAPK/ERK pathway, known to influence cell fate decisions and differentiation processes.^62^ Additionally, magnetic mechanoactivation has been demonstrated to modulate Wnt/β-catenin signaling through remote stimulation of Frizzled receptors using magnetic nanoparticles.^63^ Given that Wnt signaling plays a pivotal role in maintaining stemness and regulating OCT4 expression, it is possible that magnetic stimulation disrupts this pathway, thereby promoting differentiation. Furthermore, magnetogenetic approaches have revealed that magnetic fields can influence gene expression by altering cytoskeletal tension and nuclear architecture, which are upstream regulators of transcriptional machinery.^64^ While our data do not directly interrogate these pathways, the transcriptional shifts observed in MACrylamide ON samples suggest a convergence of mechanical and biochemical signaling, paving the way for future studies.

Overall, our results highlight the nuanced effects of dynamic 3D curvature and magnetic stimulation on cancer cell fate, emphasizing that mechanical confinement and magnetic cues can synergistically modulate cellular behavior in ways that may suppress certain malignant features despite biochemical activation.

### 2.4 Effect of the Curved Hydrogel on Healthy Dermal Cells

The final part of this study aimed to determine whether the effects of MACrylamide combined with magnetic stimulation were specific to MDA-MB-231 breast cancer cells or if similar responses could be observed in healthy cells. To address this, we used normal human dermal fibroblasts (NHDF) as a noncancerous control cell line. A Live/Dead assay was first performed following a 7-day culture on Acrylamide and MACrylamide hydrogels without magnetic exposure (Figure 4A).

**Figure 4:**
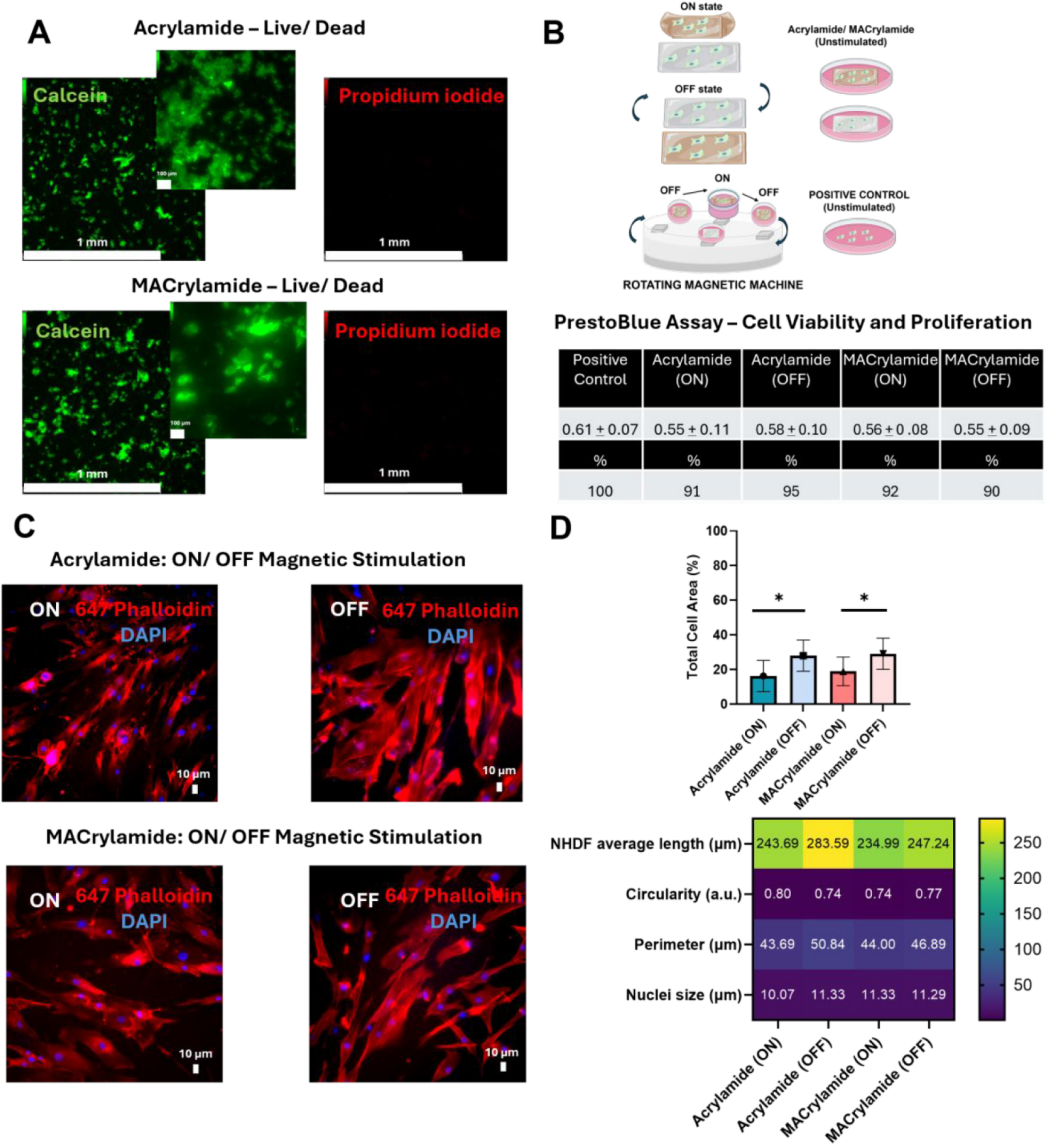
Effect of MACrylamide on healthy NHDF human dermal fibroblasts. (A) Live (green)/dead (red) imaging of dermal fibroblasts cultured on MACrylamide and Acrylamide (control condition). (B) Schematic representation of the magnetic stimulation setup and viability quantification using PrestoBlue assay (*n* = 3). (C) Fluorescence staining of nuclei (DAPI, blue) and actin cytoskeleton (Phalloidin, red) in dermal fibroblasts cultured on MACrylamide (ON and OFF) and Acrylamide (ON and OFF) at day 7. (D) Heat map of the morphological features of fibroblasts for all tested conditions (*n* = 3) and % of occupied cell area in the same conditions (*n* = 3). Data presented as means ± SD.

Calcein AM (green) was used to label viable cells, while Propidium Iodide (red) stained the nuclei of nonviable cells. The results showed predominantly viable cells in both hydrogel conditions, with no signs of cell death and no notable morphological differences between the two substrates. Given the well-documented biocompatibility of Acrylamide-based hydrogels,^27,28^ fibroblast viability on these substrates was not expected to be compromised, a conclusion further supported by the data shown in Figure 2D.

To evaluate whether the effects observed were specific to cancer cells, a similar schematic of the magnetic stimulation setup presented in Figure 3B was used for fibroblast cultures, as shown in Figure 4B. In addition to the Live/Dead assay, a PrestoBlue assay was performed to quantitatively assess cell viability and proliferation (Figure 4B). When compared to a positive control (normalized to 100% viability), fibroblasts cultured on both Acrylamide and MACrylamide showed no reduction in viability under magnetic stimulation (ON condition) relative to the nonstimulated condition (OFF). Viability remained consistently high across all groups, with no indication of cytotoxicity associated with either hydrogel or magnetic exposure. These findings confirm that MACrylamide and the magnetic actuation protocol used in this study do not adversely affect healthy dermal fibroblasts, reinforcing the specificity of the observed responses in cancer cells.

Under MACrylamide conditions, fibroblasts maintained their characteristic elongated morphology. However, a reduction in fluorescence intensity was observed under magnetic field exposure (ON condition), similar to the trend seen in MDA-MB-231 cells. Although overall viability and proliferation remained unaffected; to mirror the analysis performed on breast cancer cells, the morphological characteristics of dermal fibroblasts were also assessed using fluorescence microscopy. DAPI was used to stain cell nuclei (blue), and Phalloidin labeled actin filaments in the cytoskeleton (red) (Figure 4C). In both Acrylamide and fibroblasts exposed to magnetic stimulation appeared smaller in size. ImageJ was used to analyze several morphological parameters in this observation, including average cell length, circularity, perimeter, nuclear size, and total occupied cell area (%) (Figure 4D). A statistically significant difference was detected in the area occupied by fibroblasts across conditions, ranging from 20% to 40%. In both hydrogel types, cells exposed to the magnetic field occupied less surface area, which may partially reflect reduced fluorescence signal rather than true morphological contraction. The heat map in Figure 4D summarizes the key morphometric parameters. Despite the observed reduction in occupied area under magnetic stimulation, no other significant differences were found across conditions, suggesting that magnetic exposure induces minimal morphological changes in healthy fibroblasts.

Our findings indicate that dermal fibroblasts are less responsive to the combined effects of MACrylamide and magnetic field stimulation compared to MDA-MB-231 cells, and this finding aligns with existing knowledge that fibroblasts are generally less sensitive to mechanical stimuli than cancer cells.^65^ As key players in tissue homeostasis and wound healing,^66^ fibroblasts tend to exhibit more stable, homeostatic behaviors and may engage different signaling pathways in response to mechanical cues than those seen in transformed or malignant cells.

The observed reduction in cell area under magnetic field exposure, despite no significant changes in viability or proliferation, may reflect subtle alterations in cytoskeletal organization rather than overt changes in cell function. Magnetic fields have been shown to influence cytoskeletal organization and cell polarization, which may explain the observed morphological shifts without affecting overall cell health.^59,60^ The fact that no significant changes were observed in terms of overall proliferation or viability suggests that the magnetic field’s effect might be more subtle, influencing cell shape and potentially cell signaling rather than cell death or growth inhibition. Moreover, the lack of pronounced changes across hydrogel types and magnetic conditions implies that the specific mechanical properties of MACrylamide may not sufficiently stimulate fibroblasts within the time frame of this study. It is also possible that fibroblasts may require longer culture periods or different stimulation parameters to fully engage mechanotransductive signaling pathways. In contrast, the pronounced response of MDA-MB-231 breast cancer cells to the same stimuli underscores the heightened mechanosensitivity of cancer cells, which may be attributed to their dysregulated signaling networks and increased cellular plasticity. Tumor cells—particularly highly aggressive subtypes—often display elevated responsiveness to environmental cues, including stiffness, curvature, and external forces. This differential sensitivity highlights the importance of cellular context in shaping mechanobiological responses and supports the idea that cancer cells, unlike fibroblasts, may leverage mechanical inputs to promote malignant behavior.^66,67^

While the present study focused on early mechanotransductive events relevant to cancer initiation, future studies will investigate whether prolonged exposure to magnetic-induced curvature influences migratory or invasive behavior, to explore potential implications for tumor progression.

## 3 Conclusions

This study presents a magneto-responsive hydrogel platform MACrylamide, which enables dynamic control of curvature and surface wettability in response to external magnetic fields. Through this system, we sought to understand how physical cues such as tissue-like curvature and transient mechanical stimulation affect cancer cell behavior, with the broader goal of identifying mechanobiological features that could inform future *in vitro* cancer models and potentially therapeutic screening strategies.

By systematically comparing MDA-MB-231 breast cancer cells and healthy dermal fibroblasts on flat and curved hydrogels, with and without magnetic actuation, we uncovered striking cell-type specific responses. In cancer cells, the MACrylamide scaffold induced significant changes in morphology, gene expression, and metabolic activity. Morphological analyses revealed reduced cell spreading, increased clustering, and cytoskeletal reorganization under magnetic stimulation—features indicative of mechanical stress adaptation rather than increased aggressiveness. This was further supported by gene expression patterns: magnetic field exposure (ON) led to upregulation of stress-related markers such as NMO1 yet suppressed stemness-associated genes like Oct4 and SOX4. Conversely, in the absence of magnetic stimulation (OFF), cells on the curved scaffold exhibited higher expression of these stemness markers, suggesting that persistent curvature without mechanical agitation may create a permissive niche for cancer stem cell-like traits to emerge.

These findings point to a nuanced interplay between physical architecture and dynamic stimulation in shaping cancer cell fate. While magnetic stimulation activates biochemical stress responses, it does not appear to enhance malignancy directly—instead potentially placing cells in a more dormant or adaptive state. This highlights the importance of mechanical context: static curvature may simulate early tumor-promoting environments, while dynamic mechanical cues might disrupt the progression toward more invasive phenotypes. In contrast, dermal fibroblasts exhibited limited responses across all experimental conditions. Their morphology, viability, and gene expression remained largely stable, reinforcing the idea that nontransformed cells possess a more robust resistance to mechanical and magnetic perturbations. This differential sensitivity between cancerous and healthy cells emphasizes the utility of magnetically responsive scaffolds for isolating cancer-specific mechanobiological behaviors.

Together, our results demonstrate that MACrylamide scaffolds offer a powerful tool for dissecting how mechanical forces and dynamic topographies influence cancer biology, offering new insights into how mechanically dynamic scaffolds and magnetic actuation can modulate early cancer cell behavior. While transcriptional changes suggest potential shifts toward plasticity or altered differentiation, future studies are needed to determine whether these cues functionally enhance stemness or tumor-initiating potential.

By mimicking aspects of the tumor microenvironment—such as intermittent strain and 3D curvature—this platform reveals how malignant and healthy cells differently perceive and respond to their surroundings.

These insights not only deepen our understanding of cancer cell plasticity but also provide a foundation for developing more predictive *in vitro* models that integrate both biochemical and biophysical stimuli.

## 4 Experimental Section

### 4.1 Magnetic Nanoparticle Synthesis

The magnetic nanoparticles (MNPs) were synthesized using a coprecipitation method previously described by Olle et al.^68^ A 25 mL solution of 0.35 M FeCl_2_ (3.58 g of FeCl_2_·4H_2_O) and 0.72 FeCl_3_ (9.73 g of FeCl3·6H2O) (Sigma-Aldrich, Germany) was produced and agitated at room temperature until completely dissolved. In a nitrogen environment, a 1.0 M NH_4_OH solution was prepared in Milli-Q ultrapure water and stirred continuously at 1250 rpm. The iron salt solution was then added dropwise or using a flow rate of 5.0 mL/min using a peristaltic pump. MNPs were formed spontaneously due to the coprecipitation of the two iron slats in media with high pH. The obtained black precipitate was separated from the liquid phase using a magnetic field, then magnetically washed three times with ethanol (70% v/v) two times with phosphate-buffered saline (PBS, 100 mM sodium phosphate, 150 mM NaCl, pH 7.4). The MNPs were dried for 1 week at 37 °C in ethanol. The MNPs were analyzed by transmission electron microscopy (TEM) and an average diameter of 16.7 nm was determined with imageJ (standard deviation = 3.4 nm; Polydispersity index – PDI = 0.0395). A representative TEM image of the MNPs is shown in Figure S8, along with the frequency distribution of the diameter of the nanoparticles.

### 4.2 Fabrication of the Curved Hydrogel Using Silanization

The preparation of the curved hydrogel involved the prior preparation of silane solution in a 1:2:3 proportion of propyl methacrylate (Merck Life Science NV, Amsterdam, Netherlands), acetic acid (Sigma-Aldrich, Germany) and Milli-Q water, followed by 5 min of gentle mixing. The solution was cast in a glass slide with two stripes of tape to create specific regions where the surface of the glass was not treated with silane solution. The treated glass slide was then stored in vacuum for 2h, followed by removal of extra silane solution, as well as the tape stripes. The hydrogel was fabricated using three well glass bottom Ibidi chips (cat. no: 8038, Ibidi GmbH, Gräfelfing, Germany). A polyacrylamide hydrogel solution was prepared by mixing 40 wt % acrylamide solution, 1.8 wt % bis-acrylamide monomers with 3 mg of previously synthesized MNPs with an average size of 20 nm, as described in a previous work^20^ at a volume ratio of 1:1:1. The solution was mixed thoroughly using a magnetic stirrer and warmed up to 60 °C to ensure homogeneity. To initiate the polymerization process, a photoinitiator, 2-8 hydroxy-2-methylpropiophenone (Sigma-Aldrich, Netherlands), was added to the hydrogel solution at a volume ratio of 1:100 to achieve cross-linking. The prepared hydrogel solution was carefully pipetted into the wells of the Ibidi 3-well glass bottom chip. The hydrogel solution in the chip was exposed to ultraviolet (UV) light using a UV-LED exposure system (UV-EXP 150s, IDONUS, Switzerland) at a light intensity of 40 mJ/cm^2^ and a dose of 500 mJ/cm^2^. Afterward, the hydrogel was left to dry and shrunk overnight in the fridge at 4C, leading to a small curvature.

### 4.3 Scaffold Surface Characterization

The surface profile of Acrylamide and MACrylamide were characterized by scanning electron microscope (SEM). For this characterization, the hydrogel samples were cut into small sections and fixed onto SEM stubs. The hydrogels were imaged using SEM (FEI ESEM Quanta 600) at an acceleration voltage of 10 kV.

### 4.4 Swelling

The swelling of the hydrogels was determined through the calculation of the weight measurement overtime (at room temperature) of hydrated hydrogels of Acrylamide and MACrylamide. The swelling ratio (q) was obtained though the equation: q = W_s_/W_d_, (where W_s_ is the weight of swollen hydrogel and W_d_ is the weight of dried hydrogel) until the maximum weight of the hydrogels was achieved and stabilized.

### 4.5 Surface Wettability

Surface contact angles of the scaffolds (Acrylamide and MACrylamide) were determined using glycerol as the liquid phase in the absence and presence of a magnetic field up to 0.08 T. The magnetic field was created by a neodymium magnet placed underneath the hydrogel scaffolds. The dynamic glycerol contact angles were determined in a sessile drop mode using a drop shape analyzer system coupled to a video camera connected to a PC for data acquisition. The average contact angle values were obtained for at least three triplicates.

### 4.6 Mechanical Characterization

Compression mechanical testing was performed using circular pieces of the Acrylamide and MACrylamide hydrogels with a diameter of 8 mm in an MTS criterion tensile machine along with a confined compression setup and the data analyzed in the software TWElite to obtain load vs extension values. To verify hydrogel stiffness, the Young’s modulus of every hydrogel sample was calculated with the data collected from mechanical tests.

### 4.7 Functionalizing the Magnetic Hydrogel for Cell Culture

After the polymerization, the hydrogels were carefully rinsed for 4 cycles with deionized water to remove any unreacted monomers, photoinitiator residues, or impurities. Sterilization was performed using standard UV sterilization in a biosafety cabinet for 20 min, to ensure aseptic conditions for subsequent cell culture experiments. The hydrogels were then coated with 1% laminin solution and incubated at 37 °C for 1 h prior to starting the cell culture.

### 4.8 Breast Cancer Cell Culture on the Hydrogels

Metastatic human breast cancer MDA-MB-321 cells were cultured using high-glucose Dulbecco’s Modified Eagle Medium (DMEM, Gibco, Grand Island, New York, U.S.) supplemented with 10% fetal bovine serum (FBS, Gibco), 1% Penicilin/Streptomycin, 25 mM HEPES, 4.5 g/L d-glucose, and l-glutamine. Cells were maintained in an incubator at 37 °C, with 5% CO_2_ and 21% O_2_ in a humidified atmosphere. Cells were maintained in an incubator at 37 °C, with 5% CO_2_ and 21% O_2_ in a humidified atmosphere. Medium renewal was performed every 3–4 days. Cells were seeded on the hydrogels at a density of 300,000 cells/mL on cell culture plates with a covalently bound hydrogel layer to inhibit cellular attachment (Costar 24 ultralow attachment well plates). At day 6 after seeding the cells on the hydrogels, the samples were placed in a rotating magnetic system (under a frequency of approximately 0.2 Hz), containing 12 magnets with 1 cm^2^ and 100 mT intensity for 24 h. Afterward, the samples were fixed with PFA 4% and analyzed.

### 4.9 NDH Fibroblast Cell Culture on the Hydrogels

The human dermal fibroblasts cell line (NDHF) were cultured using high-glucose Dulbecco’s Modified Eagle Medium (DMEM, Gibco, Grand Island, New York, U.S.) supplemented with 10% fetal bovine serum (FBS, Gibco), 1% Penicilin/Streptomycin, 25 mM HEPES, 4.5 g/L d-glucose, and l-glutamine. Cells were maintained in an incubator at 37 °C, with 5% CO_2_ and 21% O_2_ in a humidified atmosphere. Medium renewal was performed every 3–4 days. Cells were seeded on the scaffolds at a density of 150,000 cells/mL on cell culture plates with a covalently bound hydrogel layer to inhibit cellular attachment (Costar 24 ultralow attachment well plates). Similarly to the experiment with breast cancer cells, on day 6 after seeding the cells on the hydrogels, the samples were placed in a rotating magnetic system (at a frequency of approximately 0.2 Hz), containing 12 magnets with 1 cm^2^ and 100 mT intensity for 24h. Afterward, the samples were fixed with PFA 4% and analyzed.

### 4.10 Lactate Dehydrogenase (LDH) and prestoblue Assay

For the LDH assay (ThermoFisher), 100 μL of supernatant was collected on day 7 from all conditions—Acrylamide and MACrylamide hydrogels with magnetic field ON and OFF, a positive control without hydrogels or magnetic exposure, and a negative control with cells incubated on latex material—using MDA-MB-231 cells. Samples were tested in duplicates and mixed 1:1 with the kit reagent (catalyst and dye solution) following the manufacturer’s instructions (50 μL sample +50 μL reagent). After incubating for 30 min in the dark, absorbance was measured at 490 nm.

For the PrestoBlue assay (Invitrogen), cells from all conditions, including a positive control without hydrogels or magnetic stimulation (day 6), were incubated on day 7 with 10% PrestoBlue reagent for 2 h at 37 °C. Fluorescence intensity was then measured at 560 nm excitation and 590 nm emission wavelengths, with fluorescence proportional to viable cell number.

### 4.11 Cell Morphology and Viability on Magnetic Scaffolds

Cells cultured on magnetic hydrogels were assessed on day 7 of the experimental time. Cells were washed twice with phosphate buffer saline (PBS, Dulbecco’s Sigma-Aldrich), and stained using LIVE/DEAD viability/cytotoxicity kit, for mammalian cells (ThermoFisher Scientific, L3224) according to the manufacturer protocol, for 20 min. Cells were imaged in a fluorescence microscope and afterward washed again twice with PBS and fixed with 4% paraformaldehyde (Sigma-Aldrich) for 20 min. Cells are permeabilized with 0.1% Triton X-100 for 10 min and incubated with Phalloidin-TRITC (Invitrogen, 1.5 μg/mL) for 20 min and 4′,6-diamidino-2-phenylindole (DAPI, ThermoFisher Scientific, 1.5 μg/mL) for 5 min, then washed with PBS for fluorescence imaging.

### 4.12 MTT Cytotoxicity Assay

The biocompatibility of the scaffolds was demonstrated through a cytotoxicity assay. NHDF dermal fibroblasts were seeded at 80,000 cells/cm^2^ in a 24-well plate and incubated for 48 h with low-glucose Dulbecco’s Modified Eagle Medium (DMEM, Gibco, Grand Island, New York, U.S.) supplemented with 10% fetal bovine serum (Gibco) and 1% antibiotic-antimycotic (Gibco) and kept at 37 °C, 5% CO_2_ and 21% O_2_ in a humidified atmosphere. Sterile scaffolds were placed on top of the fibroblast monolayer for 24 h and then observed in an EVO optical microscope for quantification of the halo formed in the area between the scaffold and the monolayer. Indirect assays were performed using latex material as the positive control and fibroblast culture media as the negative control. The lixiviates of the scaffolds incubated for 24 h to allow the release of eventual toxic substances to the media were used as a replacement for the fibroblast culture media and incubated for another 24 h. MTT [3-(4,5-Dimethylthiazol-2-yl)-2,5-Diphenyltetrazolium Bromide] solution (1 mg/mL) was prepared and replaced the lixiviates in the fibroblasts culture, for a 2 h incubation period. MTT solvent (HCl and IPA – 1:100, Sigma-Aldrich) was added to the MTT solution in the cell culture and stirred for 5 min. Absorbance was quantified at 570 nm to determine total cell viability and biocompatibility.

### 4.13 Quantitative Reverse Transcription-Polymerase Chain Reaction Analysis

The expression levels of key genes^69,70^ were quantified from the extraction of the RNA of cells for all the experimental groups. Cells were detached (either from scaffolds or TCP) and lysed, after 24 h of magnetic exposure, using consistent up and down pipetting movements for 10 min and a lysis buffer (RLT), which is part of the RNeasy Mini Kit (Qiagen, Hilden, Germany) for RNA extraction. The detachment process was aided by placing the culture plates in a stirring plate (300 rpm) for 10 min. Cell lysates were stored at −80 °C. Subsequent procedures for total RNA extraction were performed following the manufacturer’s instructions on the RNeasy Mini Kit. Complementary DNA was synthesized from 20 nm of total RNA using iScript Reverse Transcription Supermix (Bio-Rad, Hercules, California, U.S.). The reaction mixture (20 μL) was incubated in a 96-well thermal cycler (Applied Biosystems, Foster City, California, U.S.) for 5 min at 25 °C, 30 min at 42 °C and 5 min at 85 °C and then maintained at 4 °C. Gene expression levels of VEGF-A were assessed. Sequences of the specific primer sets are given in Table S2. The quantitative reverse transcription-polymerase chain reaction (qRT-PCR) was performed using SYBR Green PCR Master Mix (Applied Biosystems). All reactions were carried out at 95 °C for 10 min, followed by 40 cycles of 95 °C for 15 s and 60 °C for 1 min, according to the manufacturer’s instructions. Glyceraldehyde 3-phosphate dehydrogenase (GAPDH) was used as an internal control to normalize differences in total RNA levels in each sample. A threshold cycle (C*t*) was observed in the exponential phase of amplification, and quantification of relative expression levels was performed using standard curves for target genes and endogenous control. Geometric means were used to calculate the ΔΔC*t* values and are expressed as 2^–ΔΔC*t*^. The mean values from the triplicate analysis were compared. All conditions were tested on triplicates.

### 4.14 Statistical Analysis

All measurements were performed in triplicate under independent conditions. Results are presented as the mean ± standard deviation (SD). A two-way and one-way ANOVA with Sidak’s multiple comparisons test was used to compare the means of three independent measurements (*n* = 3). All statistical analysis was conducted using GraphPad Prism version 7 (GraphPad Software, La Jolla, CA, USA). *p < 0.05 was considered statistically significant; **p < 0.01, very significant; ***p < 0.001, highly significant; and ****p < 0.0001, extremely significant. For the RT-qPCR results of the MDA-MB-231 cells, multiple unpaired *t* tests were used for the statistical analysis using the same GraphPad Prism, where * means discovery (q < Q) while nd represents “not a discovery” (q ≥ Q).

## Supplementary Material



## Notes

Additional acrylamide and MACrylamide material characterization; curvature radius, elasticity modulus, cytotoxicity, and contact angle analysis; biocompatibility images of MDA-MB-231 cells cultured on top of acrylamide and MACrylamide scaffolds; additional images of MDA-MB-231 cells cultured on MACrylamide under ON and OFF conditions at day 7; relative gene expression of cancer-related genes for MDA-MB-231 cells cultured on polystyrene—positive control (ON and OFF); transmission electron microscopy (TEM) images of the synthesized MNPs and frequency distribution histogram; MTT assay results before and after washing cycles of acrylamide and MACrylamide; oligo names and sequences of the genes used in this study (PDF)
