# Supplementary material for: Curved Magnetic Hydrogels for Understanding Cancer Initiation
Source: ACS Appl Mater Interfaces. 2025 Aug 28;17(36):50364–77. doi: 10.1021/acsami.5c11408 (PMC12442011; doi:10.1021/acsami.5c11408)
Supplement: Supplementary file 1 [file am5c11408_si_001.pdf]

Supporting Information

Curved magnetic hydrogels for understanding cancer initiation

Ana C. Manjua[1,2*], Christian M. Verkerk[1], Burcu Gumuscu[1,2,3*]

[1]Biosensors and Devices Lab, Department of Biomedical Engineering, Eindhoven University of Technology, 5600 MB, Eindhoven, The Netherlands

[2]Institute of Complex Molecular Systems, Eindhoven University of Technology, 5600 MB, Eindhoven, Netherlands

[3]Eindhoven Artificial Intelligence Systems Institute, Eindhoven University of Technology, 5600 MB, Eindhoven, The Netherlands

***Corresponding authors:**
Ana C. Manjua: a.c.baeta.manjua@tue.nl
Burcu Gumuscu: b.gumuscu@tue.nl

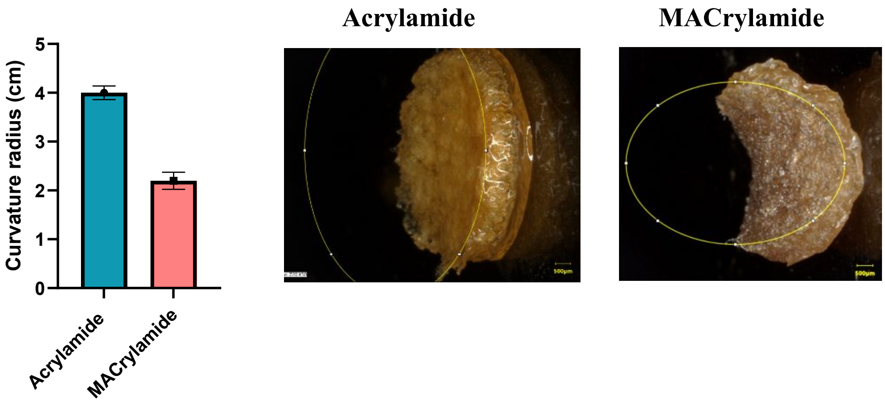

Figure S1. Curvature radius of the acrylamide and MACrylamide under magnetic actuation using Fit circle plugin from ImageJ (n=5). Data presented as mean ± SD.

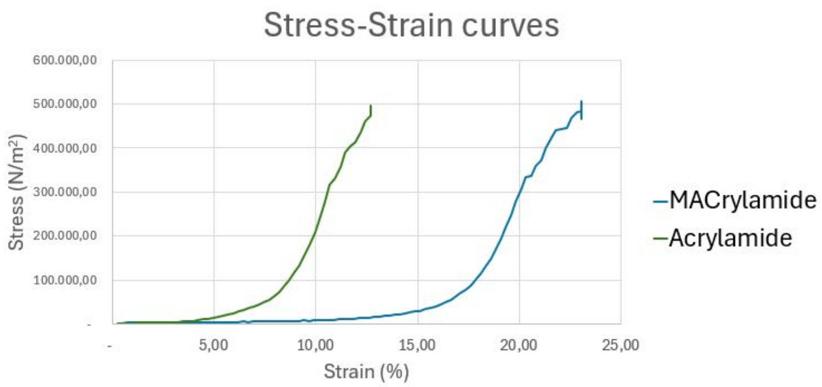

Figure S2. Compressive test reporting the representative characterization curves for Acrylamide and MACrylamide hydrogels.

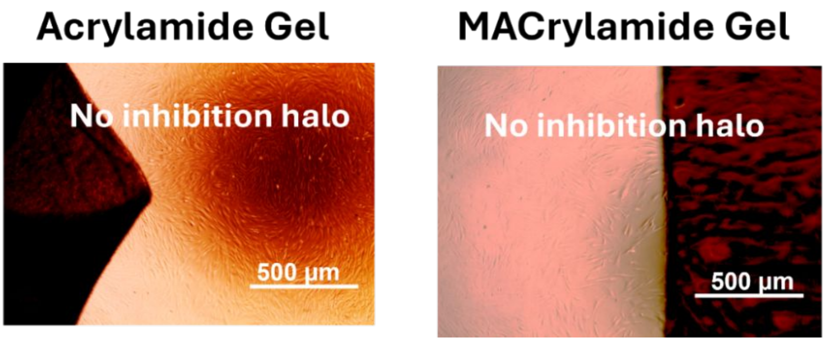

Figure S3. Direct contact of the hydrogels with fibroblasts to assess the formation of inhibition halo due to cytotoxic behavior.

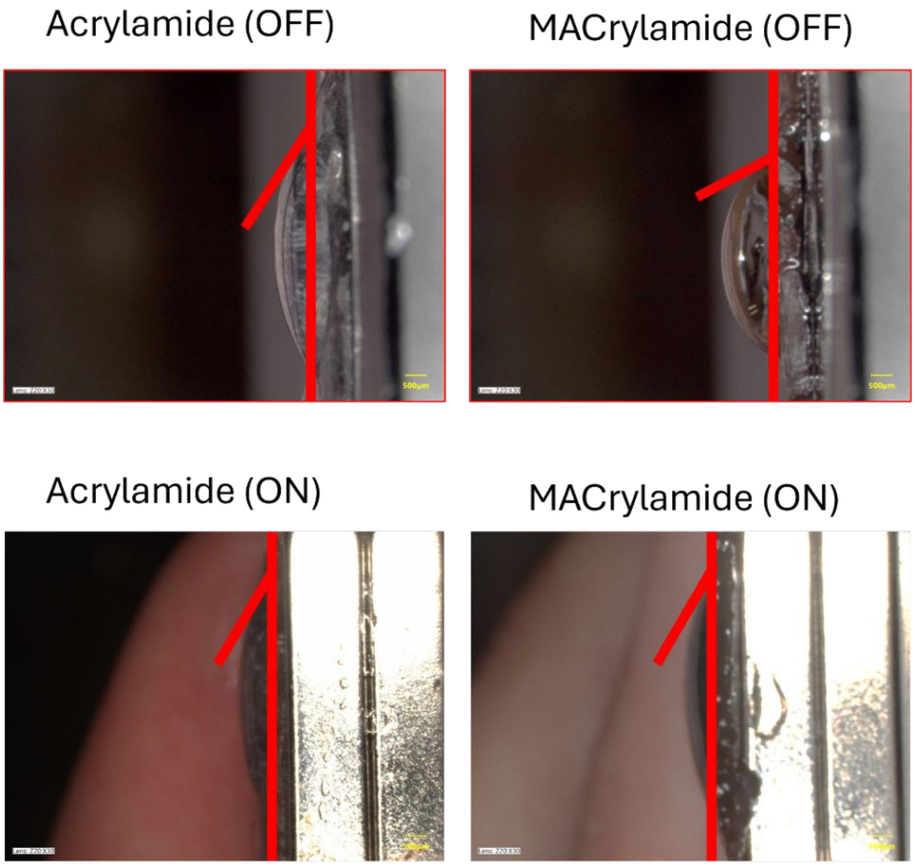

Figure S4. Contact Angle of the hydrogels with (ON) and without (OFF) magnetic exposure during the measurements.

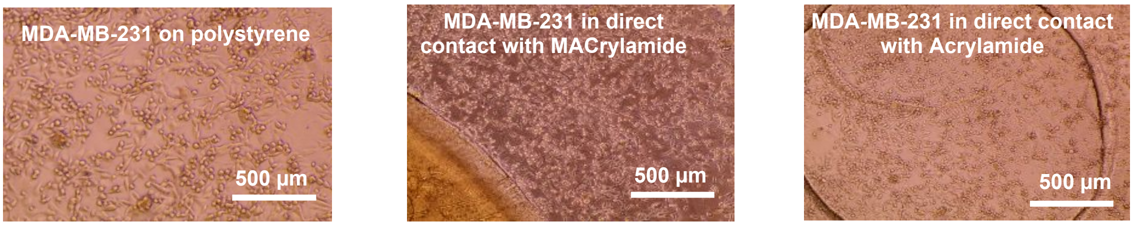

Figure S5. Biocompatibility of MACrylamide with MDA-MB-231 cells in direct contact with the hydrogel and comparing behavior on standard polystyrene surfaces.

MAGNETIC FIELD ON

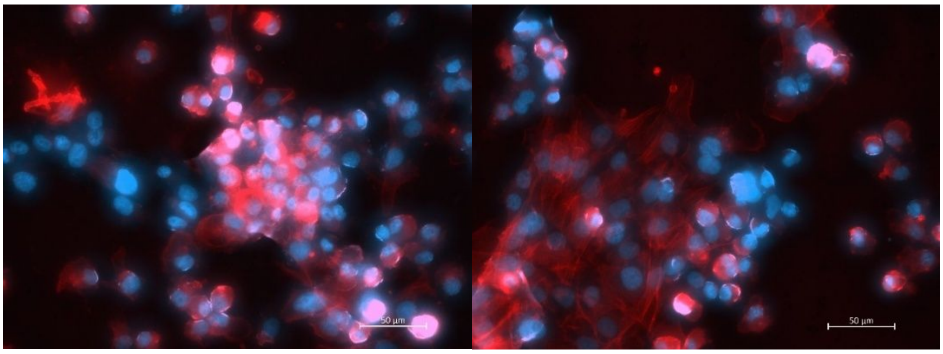

MAGNETIC FIELD ON (using ZEISS apotome microscope)

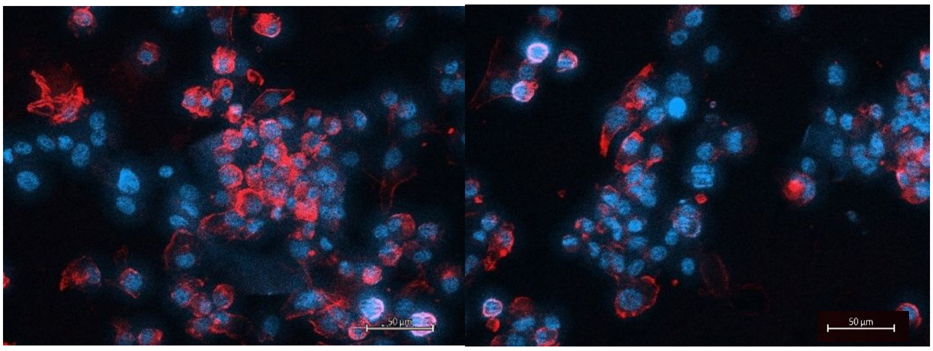

MAGNETIC FIELD OFF

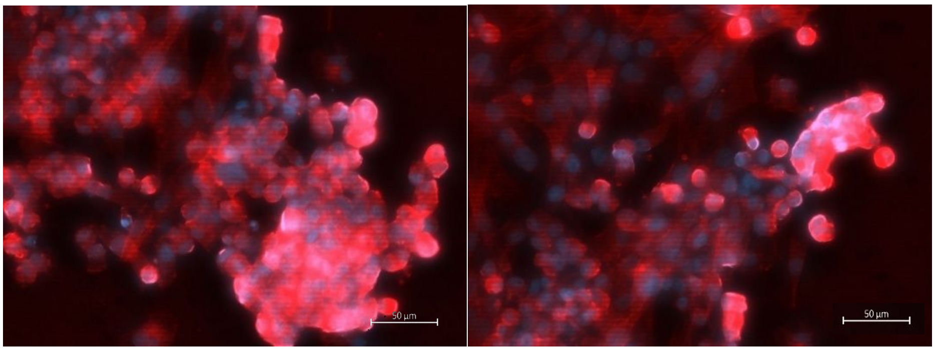

MAGNETIC FIELD OFF (using ZEISS apotome microscope)

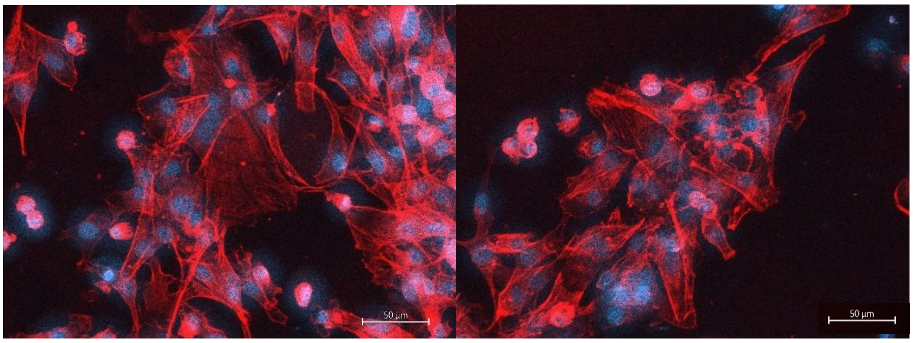

Figure S6. Additional images of MDA-MB-231 cultured on MACrylamide under ON and OFF conditions at the final day of the experiment (day 7).

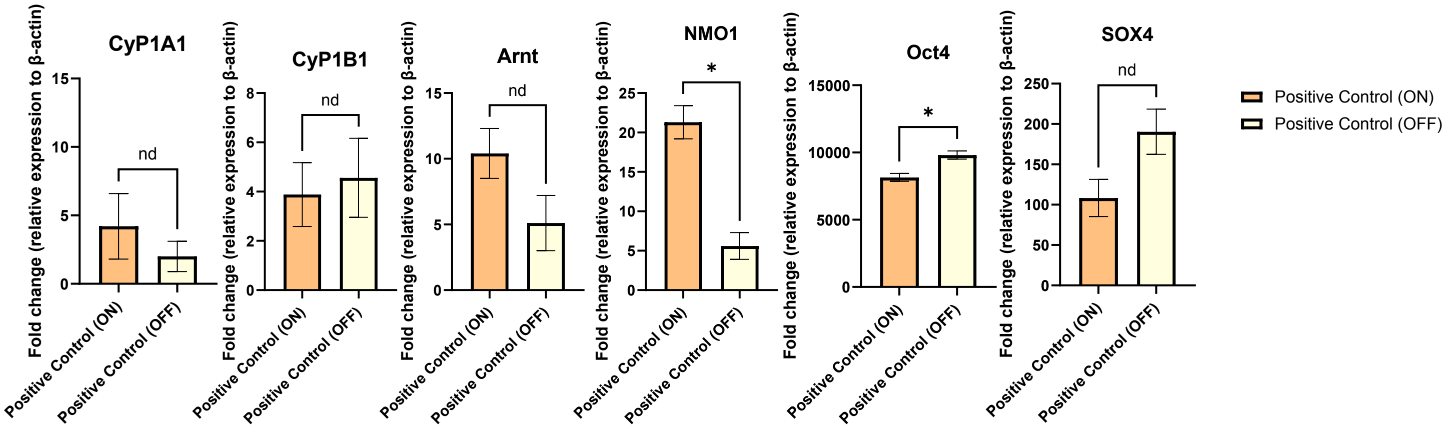

Figure S7. Relative gene expression of cancer-related genes for MDA-MB-231 cells cultured on polystyrene - Positive Control (ON and OFF) on day 7 (n = 3). Data presented as means ± SD.

A **B**

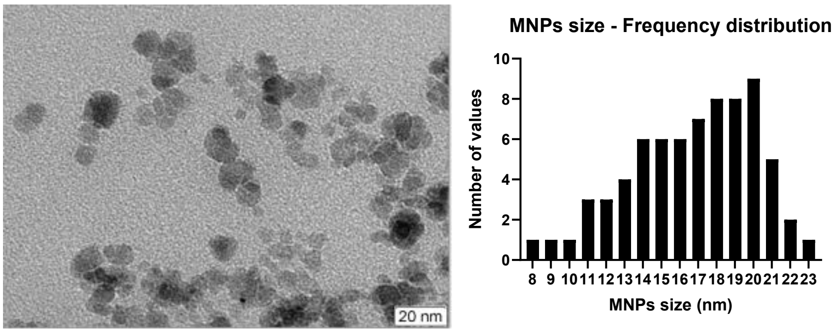

Figure S8. (A) Transmission electron microscopy (TEM) image of the synthetized MNPs. **(B)** Frequency distribution (histogram) of the MNPs diameters measured by ImageJ.

Table S1. Cytotoxicity results with MTT assay before and after cycles of the washing of the hydrogels (Acrylamide and MACrylamide) to release unbound compounds.

- MTT assay (Before washing procedures on the hydrogels)

Positive Control (Cell media)	Negative Control (Latex)	Acrylamide	MACrylamide (3%)	MACrylamide (1%)	MACrylamide (0.5%)
0,131	0,043	0,048	0,071	0,078	0.080
0,137	0,043	0,049	0,076	0,075	0.083
0,132	0,045	0,047	0,080	0,070	0.082
**Average**	**Average**	**Average**	**Average**	**Average**	**Average**
0,132	0,043	0,048	0,076	0,074	0.082
**%**	**%**	**%**	**%**	**%**	**%**
100	33	36	58	56	62

- MTT assay (After 4 cycles of hydrogels washing)

Positive Control (Cell media)	Negative Control (Latex)	Acrylamide	MACrylamide (3%)	MACrylamide (1%)	MACrylamide (0.5%)
0,106	0,053	0,150	0,124	0,121	0,130
0,112	0,059	0,107	0,120	0,118	0,129
0,123	0,051	0,115	0,128	0,115	0,134
**Average**	**Average**	**Average**	**Average**	**Average**	**Average**
0,114	0,054	0,124	0,124	0,118	0,131
%	%	%	%	%	%
100	46	110	110	104	115

Table S2. Oligo names and sequences of the genes used in this study.

Oligo Name	Sequence
Beta-actin_F	GTGGGGCGCCCCAGGCACCA
GAPDH	GGTCACCAGGGCTGCTTTTA
Beta-actin_R	CTCCTTAATGTCACGCACGATTTC
CYP1A1_F	TAGACACTGATCTGGCTGCAG
CYP1A1_R	GGGAAGGCTCCATCAGCATC
CYP1B1_F	AACGTCATGAGTGCCGTGTGT
CYP1B1_R	GGCCGGTACGTTCTCCAAATC
Arnt_F	CGGAACAAGATGACAGCCTAC
Arnt_R	ACAGAAAGCCATCTGCTGCC
NMO1_F	GGAGAGGCTGGTTTGAGCGAGTGTTC
NMO1_R	ATTTGAATTCGGGCGTCTGCTG
OCT4_F	CAATTTGCCAAGCTCCTAAA
OCT4_R	TTGCCTCTCACTTGGTTCTC
Sox4_F	TGATACGGTAGGAGCTTTGC
Sox4_R	GGTCTCTAAAGGGGCAAAAG
